# Aging Initiative: A Student-Led Model for Advancing Aging Research with a Translational Focus

**DOI:** 10.1093/geroni/igaf122.3536

**Published:** 2025-12-31

**Authors:** Maks Fedorovskyy, Satvik Dasariraju, Sean Simonini, Eshan Mehra, Chancen Law, Nina Khera, Krishna Rajagopal, Ariela Orkaby

**Affiliations:** Harvard University, Cambridge, Massachusetts, United States; Harvard University, Cambridge, Massachusetts, United States; University of Massachusetts Lowell, Lowell, Massachusetts, United States; Harvard University, Cambridge, Massachusetts, United States; Harvard University, Cambridge, Massachusetts, United States; Harvard University, Cambridge, Massachusetts, United States; Harvard University, Cambridge, Massachusetts, United States; Brigham & Women’s Hospital, Harvard Medical School, Boston, Massachusetts, United States

## Abstract

Aging research is advancing rapidly but faces a striking shortage of young talent; new educational frameworks are needed to equip future generations with a “full pipeline” viewpoint (spanning research, drug development, and policy) required to bring new science to the clinic. The Aging Initiative is a new undergraduate-led nonprofit, representing a scalable model for student-driven efforts to propel the field. By equipping students and early-career researchers with direct mentorship and hands-on interdisciplinary opportunities in research, biotechnology, and policy, we aim to dismantle barriers that hinder translation. Founded at Harvard in 2023 with seven members, the organization has grown in two years into a Boston-wide community of over 1700 students, researchers, and industry professionals. This rapid growth underscores strong interest and the need for structures that direct it toward lasting impact. Our programs are intentionally designed to integrate disciplines, bridge academia-industry gaps, and confront historically overlooked challenges in therapeutic development for age-related disease. In the preceding months, we hosted a 400-participant muscle aging symposium with world experts on frailty and patient care, biotech founders, and pharmaceutical company executives. In addition to a steady calendar of journal clubs, mixers with hundreds of attendees, and research seminars, we launched collaborations with partners including the Pepper Center, Biolearn, and Nucleate, spanning clinical development, biomarker analysis, and education. With upcoming events poised to bring together thousands, the Aging Initiative’s approach provides a replicable blueprint for student-driven engagement that institutions worldwide can adopt to accelerate progress and catalyze the next generation of leaders in aging science.

